# Waning success: a 2013–2022 spatial and temporal trend analysis of malaria in Ethiopia

**DOI:** 10.1186/s40249-024-01259-4

**Published:** 2024-12-09

**Authors:** Abdollah Jalilian, Galana Mamo Ayana, Temesgen Ashine, Elifaged Hailemeskel, Yehenew Asmamaw Ebstie, Eshetu Molla, Endashaw Esayas, Nigatu Negash, Abena Kochora, Muluken Assefa, Natnael Teferi, Daniel Teshome, Alison M. Reynolds, David Weetman, Anne L. Wilson, Birhanu Kenate, Martin J. Donnelly, Luigi Sedda, Endalamaw Gadisa

**Affiliations:** 1 Lancaster Ecology and Epidemiology Group, Lancaster Medical School, Lancaster University, Lancaster, UK; 2 Malaria and Neglected Tropical Disease, Armauer Hansen Research Institute, Addis Ababa, Ethiopia; 3 Public Health Emergency Management, Research, and Blood Bank Service Directorate, Oromia Region Health Bureau, Addis Ababa, Ethiopia; 4 Public Health Emergency Management, Research, and Blood Bank Service Directorate, Dire Dawa City Administration Health Bureau, Dire Dawa, Ethiopia; 5 Department of Vector Biology, Liverpool School of Tropical Medicine, Pembroke Place, Liverpool, UK

**Keywords:** *Anopheles stephensi*, Environmental factors, Malaria risk, Spatiotemporal, Ethiopia

## Abstract

**Background:**

Despite consecutive decades of success in reducing malaria transmission, Ethiopia went off track towards its goal of malaria elimination by 2030, as outlined in the NMCP malaria strategy. Recent malaria outbreaks in Ethiopia are attributed to the emergence and spread of diagnostic and drug-resistant *Plasmodium falciparum*, increased insecticide resistance in major vectors and the spread of invasive *Anopheles stephensi*. The effects of the COVID-19 pandemic, environmental anomalies and internal conflicts have also potentially played a role in increasing malaria transmission. This study aimed to evaluate the contribution of environmental factors and *An. stephensi* to the spatiotemporal trends of recent malaria cases in Ethiopia.

**Methods:**

Clinical malaria case data reported weekly between January 2013 and January 2023 were obtained from the Ethiopian Public Health Institute (EPHI), Addis Ababa. A negative binomial regression model was used to explain the variability and potential overdispersion in the weekly number of malaria cases reported across Ethiopian administrative zones. This model incorporated fixed effects for selected environmental factors and random effects to capture temporal trends, zone specific seasonal patterns, spatial trends at the zone level, and the presence of *An. stephensi* and its impact.

**Results:**

Our negative binomial regression model highlighted 56% variability in the data and slightly more than half (55%) was due to environmental factors, while the remainder was captured by random effects. A significant nationwide decline in malaria risk was observed between 2013 and 2018, followed by a sharp increase in early 2022. Malaria risk was higher in western and northwestern zones of Ethiopia compared to other zones. Zone-specific seasonal patterns, not explained by environmental factors, were grouped into four clusters of seasonal behaviours. The presence of *An. stephensi* was not shown to have any significant impact on malaria risk.

**Conclusions:**

Understanding the spatial and temporal drivers of malaria transmission and therefore identifying more appropriate malaria control strategies are key to the success of any malaria elimination and eradication programmes in Ethiopia. Our study found that approximately 50% of malaria risk variability could be explained by environmental, temporal, and spatial factors included in the analysis, while the remaining variation was unexplained and may stem from other factors not considered in this study. This highlights the need for a better understanding of underlying factors driving local malaria transmission and outbreaks, to better tailor regional programmatic responses.

**Graphical Abstract:**

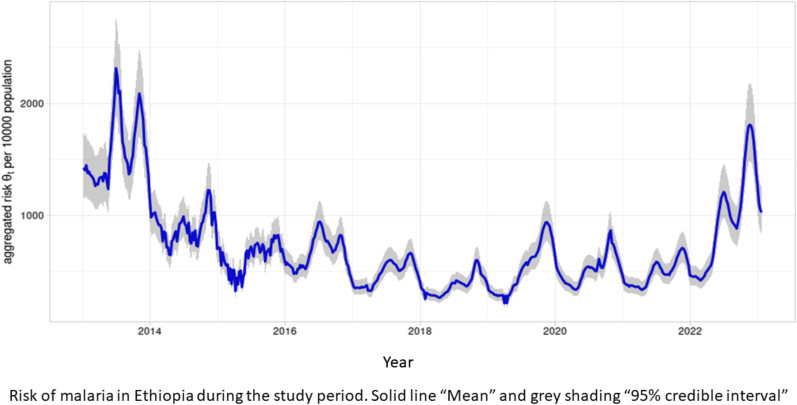

**Supplementary Information:**

The online version contains supplementary material available at 10.1186/s40249-024-01259-4.

## Background

Global efforts towards malaria control and elimination were heightened following the launch of Roll Back Malaria programme in 1998 and the declaration of Abuja in 2000, with the target of reducing malaria-related deaths by 50% before the end of 2010 [1]. Since then, investments have been made to promote universal coverage of insecticide-treated mosquito nets (ITNs), diagnostic testing, and updated malaria treatment guidelines [2]. These efforts saw a drop in malaria cases and deaths from 238 million and 736,000 in 2000 to 229 million to 409,000 in 2019, respectively [3]. Between 2000 and 2015, eight countries eliminated malaria and many others reduced transmission to low levels [4]. Motivated by these achievements, in 2015 WHO developed a global technical strategy (GTS) for 2016–2030 [2] to reduce malaria related mortality and incidence by at least 90% by 2030 from the 2015 baseline [4].

However, between 2015 and 2019, the global rate of decline was decidedly lower, at around 2% [5], highlighting that the programme might fall short of the targets for 2030 [6]. Importantly, the 2023 WHO malaria report showed that the global case incidence was off course by 55% [7]. Factors contributing to this discrepancy include the emergence of drug and diagnostic-resistant parasites [8, 9], mosquito resistance to insecticides [7, 10], the COVID-19 pandemic [10], the global rise in temperatures [7], expansion of *Anopheles stephensi* [8, 11], and weakening of control programmes due to conflict and internal population displacement [12].

In line with global momentum, in 2004, Ethiopia began scaling up interventions to prevent and control malaria, including the distribution of insecticide treated nets (ITNs), indoor residual spraying (IRS), and the introduction of rapid diagnostic tests and artemisinin-based combination therapies [13, 14]. Moreover, the ‘test-and-treat’ policy was implemented in 2010 [13]. This achieved a reduction in malaria-related deaths of 54% between 2000 and 2016 [15], and Ethiopia was one of four countries that were on course to meet the GTS target by 2020 [16].

With these gains, Ethiopia set a goal to achieve zero indigenous malaria cases in 565 and 1046 districts by 2025 and 2030, respectively [17]. To support these goals, the risk of malaria at the district level was stratified into five districts based on annual parasite incidence (API, cases/1000 people per year), altitude and expert opinions: high risk (API ≥ 50), medium risk (API ≥ 10 & < 50), low risk (API > 5 & < 10), very low risk (API > 0 & ≤ 5), and malaria-free areas (API = 0) [14]. However, efforts to prioritize elimination were complicated by a rapid growth in malaria in 2022 with an estimated 1,732,562 malaria cases [7], nearly a two-fold increase from the 2018 baseline of 962,087 [16]. Factors attributed to the nationwide upsurge included climatic anomalies [18], biological threats including *An. stephensi* [11, 19] and emergence of drug and diagnostic-resistant *Plasmodium falciparum* (*P. falciparum)* [8, 9], service interruptions due to the COVID-19 pandemic [14], and widespread internal conflicts [7].

To evaluate risk factors associated with the spatial and temporal patterns of malaria risk in Ethiopia, we therefore utilised a geospatial modelling technique [20]. Our approach can support decision-makers in tailoring interventions at a local scale and to inform technologies, strategies, and target populations [21].

## Methods

### Background to the study

Ethiopia is characterized by *Kola* or hot lowlands, *Weyna Dega*, and *Dega* or cool highlands with altitudes of ≤ 1500 meters above sea level (masl), 1500–2400 masl, and > 2400 masl, respectively. Rainfall is strongly correlated with altitude and therefore varies significantly across the country [22]. This has resulted in heterogeneous malaria transmission in a bimodal pattern [14]. The bimodal seasonal pattern of malaria transmission is mainly associated with rainfall. In most parts of Ethiopia the major transmission peaks occur from September to December following the main rainy season (June to August) and small peaks from April to June following the minor rainy season (March to May) [22, 23]. This seasonal pattern includes a lag phase of approximately 1 month following the end of the rainy season [24]. Altitude is also a major driver of malaria transmission and areas that lie below 2000 masl, where approximately 60% of Ethiopians reside, are considered malarious [17], [25]. Overall, *Plasmodium falciparum* and *Plasmodium vivax* are the co-endemic species in different proportions with a national average of 65% and 35% of all cases respectively [14].

### Malaria case data

The Ethiopia Public Health Institute (EPHI) [26] is responsible for collecting data related to public health emergency management (PHEM) [26]. The public health institute analyses weekly PHEM data on diseases that are classified as public health threats in Ethiopia, including malaria, to identify areas of concern and respond with appropriate measures. PHEM data collected at the health facility level is aggregated by catchment, district, zone, and region.

Clinical malaria cases PHEM data between January 2013 to January 2023 were obtained from EPHI. Data was stratified to the finest spatial resolution available at woreda level, the third administrative division in Ethiopia. In the Ethiopian health system, a primary hospital is expected to serve a woreda with an average population size of 60,000 to 100,000. However, significant administrative border changes were implemented between 2013 and 2022. Therefore, to reduce spatial uncertainties as a result of these changes, data were aggregated to zone level, the second administrative division which was less affected. Data completeness improved over time; the annual national rate of missing records ranged from 17.6% in 2015 to 5% in 2021. Ten zones had no missing records and the average rate of missing records was approximately 5% in all other zones. West Gondar zones had the highest missing rates (40% to 79% of records) potentially due to internal conflict.

To facilitate georeferencing of weekly malaria surveillance data we utilised the 2021 Ethiopia administrative division shapefile and its associated population size provided by the United Nations Office for the Coordination of Humanitarian Affairs [27, 28]. This shapefile includes 13 regions and 92 zones. However, to align with the recording structure of the malaria surveillance data, East Bale zone and Bale zone, Dire Dawa rural zone and Dire Dawa urban zone were all merged within the retrieved shapefile. Therefore, all analyses in this study are conducted exclusively on 90 zones in 13 regions of Ethiopia (Supplementary Table 1).

### Risk factors

Remote sensing data for Ethiopia across the whole study period were retrieved from open repositories (Table 1). Spatial and temporal resolutions of environmental factors varied across some variables. This can introduce bias due to a mismatch between the scale of change in each variable and the scale of measurement. To address this, we aggregated the environmental factors to match the spatial and temporal scale of the case counts. Specifically, for original spatial and temporal scales, we applied spatial aggregation to all environmental factors at zone level and temporal aggregation at weekly level. This aggregation process involved the computation of summary statistics, including mean, minimum, maximum and standard deviation for each variable per zone and per week. The only exception was land cover, where we calculated percentage per zone for the ten categories of land cover.

**Table 1:** Variables used for contributing factors to spatial and temporal variation in weekly malaria cases across Ethiopian zones

Variable	Code	Unit	Source	Resolution
Horizontal easterly wind speed, at a height of ten metres above the Earth’s surface	u10	Metres per second	ERA5-Land hourly reanalysis data, Copernicus's Climate Data Store, by European Centre for Medium-Range Weather Forecasts. These data are processed retrospectively using meteorological models and ground data assimilation methods	Temporal: hourly 1950–present Spatial: 1100 m
Horizontal northernly wind speed, at a height of ten metres above the Earth’s surface	v10	Metres per second		
Leaf area index, high vegetation (evergreen trees, deciduous trees, mixed forest/woodland, and interrupted forest)	lai_hv	Square meter per square meter		
Leaf area index, low vegetation (crops and mixed farming, irrigated crops, short grass, tall grass, tundra, semidesert, bogs and marshes, evergreen shrubs, deciduous shrubs, and water and land mixtures)	lai_lv	Square meter per square meter		
Skin (Earth surface) temperature	skt	Kelvin		
Total precipitation	tp	Meter		
Volume of water in soil layer 1 (0–7 cm)	swvl1	cubic meter per cubic meter		
Gridded population density	pop_density	Number of people per square kilometer	Socioeconomic Data and Applications Center, NASA, Version 4	Temporal: Year 2020 Spatial: 1000 m
Land cover	land_cover	–	CCI Land Cover (LC) team, European Space Agency	Temporal: Year 2016 Spatial: 20 m
Elevation	Elevation	Meter	U.S. Geological Survey	Temporal: Year 2007 Spatial: 450 m
Presence of invasive species	Invasive	Binary (presence/absence)	WHO Global Malaria Programme	Temporal: yearly Spatial: zone-level

To assess the impact of *An. stephensi*, we combined data on 114 mosquito sampling attempts across Ethiopia between 2016 and 2023 from the WHO malaria threats map dataset [29] and other recent fieldwork [11] (Table 1).

### Modelling of spatiotemporal variations

Although the Poisson model is commonly used for spatial and spatiotemporal count data, it assumes that the mean and variance are equal, which can be restrictive. In contrast, the negative binomial model allows the variance to be greater than the mean, a condition called overdispersion. When data shows more variability than expected under a Poisson model, the negative binomial model is a more suitable choice [30]. To address variability and potential overdispersion in the weekly number of malaria cases across Ethiopian zones, denoted by ${y}_{i,t}$ where $i$ is the zone and *t* the week, a negative binomial regression model was chosen over a Poisson model to examine the spatiotemporal relationships of malaria with the risk factors, denoted by ${x}_{i,t}=\left({x}_{i,t,1},\dots ,{x}_{i,t,M}\right)$, where *M* is the total number of risk factors. The mean of the distribution of ${y}_{i,t}$ is:

$${\mu }_{i,t}=E\left[{y}_{i,t}\right]={P}_{i,t}\times {\theta }_{i,t}$$

and variance is:

$${\sigma }_{i,t}^{2}=Var\left({y}_{i,t}\right)={\mu }_{i,t}\times \left(1+\frac{{\mu }_{i,t}}{\varphi }\right).$$

here ${P}_{i,t}$ and ${\theta }_{i,t}$ denote the known exposed population size and expected relative risk of malaria infection in the zone $i$ and week $t$, respectively, and $\varphi>0$ is the dispersion parameter. As a common approach in disease mapping with aggregated areal count data, including the population size ${P}_{i,t}$ as a multiplicative offset term to model the mean, allows adjustment for population variations. Thus, the risk ${\theta }_{i,t}$ represents the impact of all other factors besides population fluctuations [31] and is represented by a log-linear model:

$${\theta }_{i,t}=\text{exp}\left(\alpha +{f}_{i,t}+{r}_{i,t}\right)$$

where:

- Intercept: $$\alpha$$
  o expresses the unmodelled overall country-wide average of expected risk of malaria;
- Fixed effect of environmental factors: ${f}_{i,t}={\sum }_{j=1}^{M}{\beta }_{j} {x}_{i,t,j}$
  o each environmental factor ${x}_{i,t,j}$ has its own coefficient ${\beta }_{j}$;
- Random effect of other sources of variations: ${r}_{i,t}={\tau }_{t}+{s}_{i,t}+{\xi }_{i}+{\nu }_{i,t}$
  o Represents the additive combination of temporal trend, zone-specific seasonality, zone-level spatial trend and effect of invasive species, as summarised in Table 2.

As a common model in the context of disease mapping and epidemiology, a Besag-York-Mollié (BYM) model was considered for possible spatial heterogeneity and dependence between zones. This model includes two components, enabling it to capture both spatial autocorrelation among zones and heterogeneity across different zones, even after accounting for environmental factors and existing spatial autocorrelation.

**Table 2:** Random effect terms included in the log-linear model for the weekly risk of malaria in Ethiopian zones

Term	Notation	Representing	Probability distribution	Parameters (variances)
Temporal trend	${\tau }_{t}$	Overall long-term and large-scale temporal variation in the entire Ethiopia (not by zone)	First-order random walk	${\sigma }_{\tau }^{2}$
Zone-specific seasonality	${s}_{i,t}$	Recurring seasonal variations in each zone not explained by environmental factors	Cyclic second-order random walk temporal pattern and spatially exchangeable	${\sigma }_{s}^{2}$
Zone-level spatial trend	${\xi }_{i}$	Spatial variations between zones arise from dependencies or heterogeneity that are not explained by environmental factors	Besag-York-Mollié (BYM)	${\sigma }_{\xi ,Besag}^{2}$ and ${\sigma }_{\xi ,iid}^{2}$
Invasive species	${\nu }_{i,t}$	Presence of *An. stephensi* in a zone from time *i*	Independent and identically distributed	${\sigma }_{\nu }^{2}$

A Bayesian approach with integrated nested Laplace approximation method [32] was used for parameter estimation and statistical inference of the proposed model. The missing weekly counts for some zones were considered to be missing completely at random. This means that the likelihood of missing data is the same for all observations, regardless of any specific characteristics or values. Based on this assumption, any analysis performed on the available data remains unbiased, as the missingness does not systematically influence the results. All the computations for this approach were implemented through the R package INLA [33, 34]. Full computational details are provided in Gómez-Rubio (2020) [35].

To evaluate spatial patterns in the residuals of the models (which can support investigation of additional factors driving malaria) a hierarchical cluster analysis was employed to group similar zones based on their estimated random effects. Among various methods, we used the complete linkage method to calculate the distance between clusters [36]. In this approach, the distance between two clusters is defined as the maximum distance between any pair of spatial random effects from the zones within each cluster.

## Results

### Progress in malaria control and elimination efforts has stalled since 2021

**Fig. 1:**
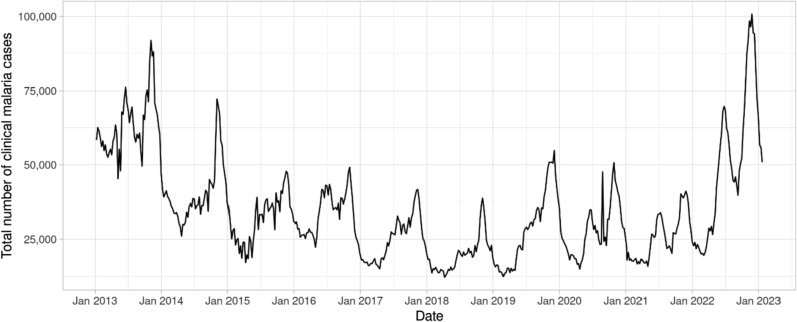
Weekly clinical malaria cases in Ethiopia from January 2013 to January 2023, EPHI

There were temporal variations in the weekly count of clinical malaria cases in the country during the study period, January 2013 to January 2023 (Fig. 1). Most years exhibited intra-annual bimodal seasonal patterns and a long-term temporal trend that declined between 2013 to 2018, and then increased from 2021 onwards. Seasonal peaks tended to occur in the second half of the year and notable variability among different regions as evident in the summary statistics of the weekly counts of clinical malaria cases by year and by region, respectively, provided in Supplementary Tables 1 and 2.

**Fig. 2:**
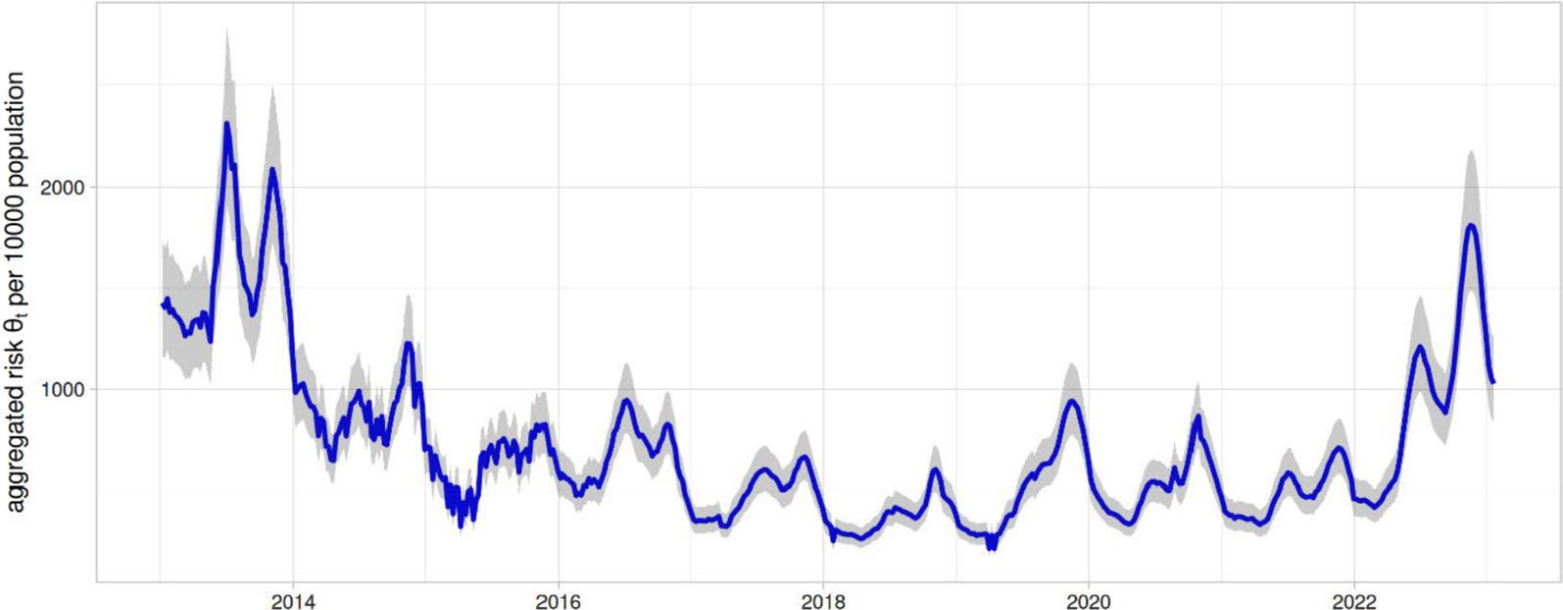
Mean (solid line) and 95% credible interval (grey shading) of the aggregated country-wide risk of malaria per 10,000 population in Ethiopia between January 2013 and January 2023, EPHI

Binomial model estimates of the malaria risk across different weeks and zones (in 90 zones); mean (lines) and associated 95% credible intervals (grey shading) for the aggregated country-wide risk of malaria cases, ${\theta }_{t}={\sum }_{i=1}^{90}{\theta }_{i,t}$, per 10,000 population (i.e. $\text{10,000}\times {\theta }_{t}$), over the study period revealed declining risk from 2013 to 2018 followed by a sharp increased risk from early 2021 (Fig. 2).

**Fig. 3:**
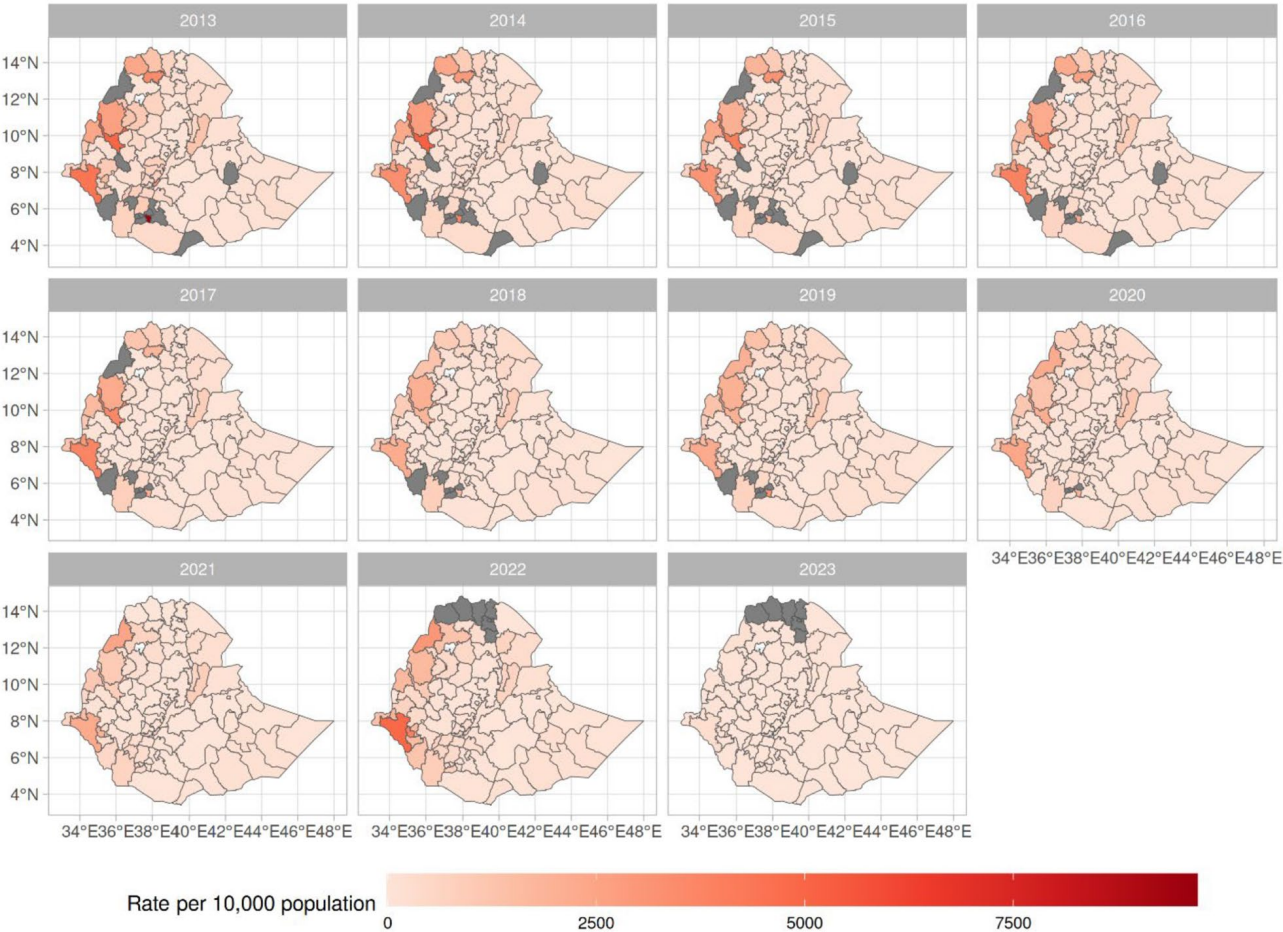
Rates of clinical malaria cases per 10,000 population across zones of Ethiopia between 2013 and 2023. Grey zones indicate no recorded cases. Lakes are represented in white. For 2022 and 2023, records were missing for North Ethiopia (West Gondar) was due to internal conflict

The spatial pattern of total clinical malaria cases per 10,000 population across zones of Ethiopia reveals a higher concentration of clinical malaria cases in western and northwestern zones (Fig. 3).

**Fig. 4:**
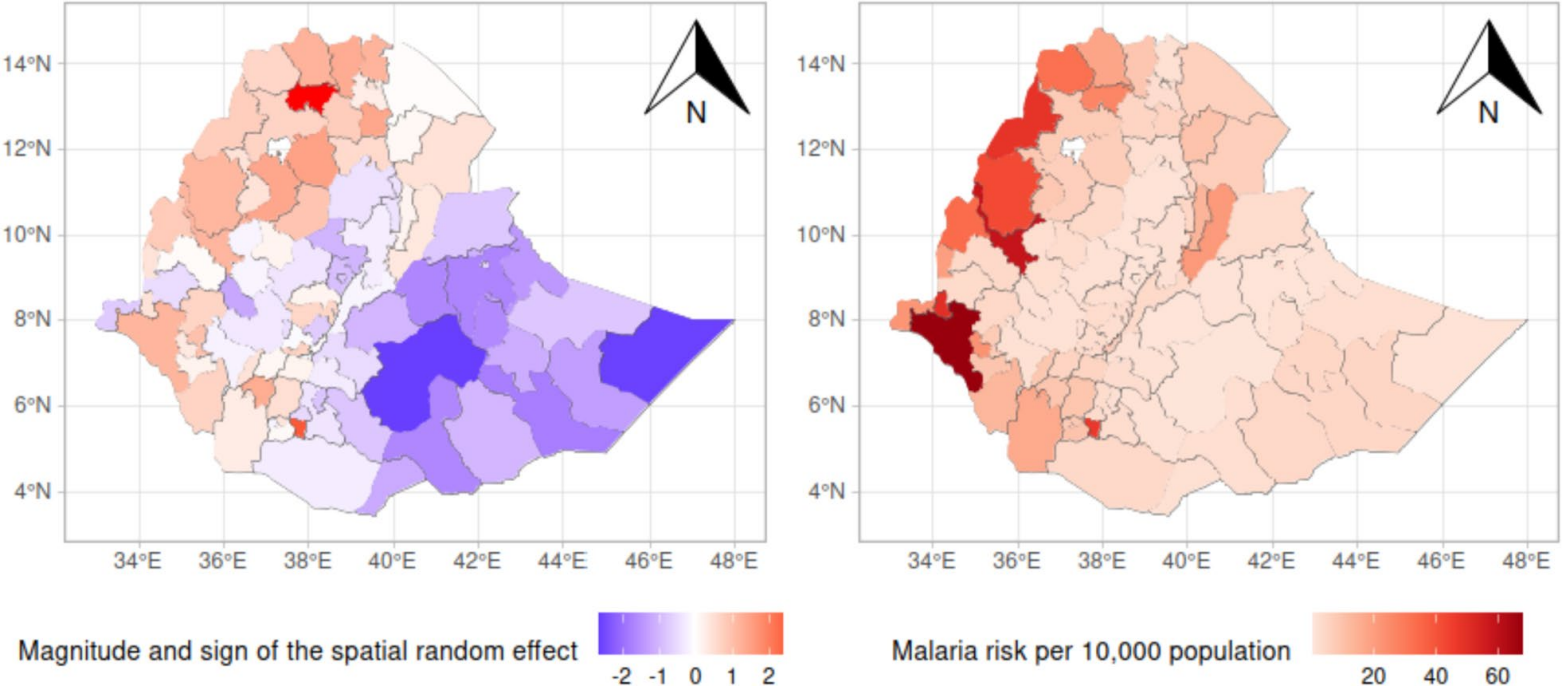
Means of the spatial random effect (left) and the estimated mean risk of malaria per 10,000 population [37] across zones of Ethiopia

The estimated risk of malaria cases per 10,000 population, $\text{10,000}\times \frac{1}{525}{\sum }_{t=1}^{525}{\theta }_{i,t}$, showed higher risk in west and northwest zones, compared to other zones. at the zone level for the 525 weeks, accounted for the overall effect of environmental factors as well as spatial and temporal random effects (Fig. 4, right panel). The means of the spatial random effect (Fig. 4 left panel), represent spatial fluctuations not accounted for by other components in the model. The sign and magnitude of these means indicate a general trend: negative spatial effects in the eastern zones and positive spatial effects in the western and northwestern zones. This suggests that predictions based solely on the fixed effects of environmental factors may underestimate or overestimate the risk of malaria. Specifically, underprediction occurs in the West, while overprediction occurs in the East.

### Approximately 50% of variability in malaria trends over the last decade, as captured by the model, can be attributed to environmental factors

A goodness-of-fit measure (Bayesian R-squared) indicated that approximately 56% of variation in the weekly number of clinical malaria cases in Ethiopia is explained by the model. Of this explained variation, 55% is attributable to environmental factors.

In terms of the importance of the different residual model components (therefore excluding fixed effects), the zone-specific seasonal random effect ${s}_{i,t}$ and spatially structured part of the spatial random effect ${\xi }_{i}$ are components with larger variances and therefore contribute more to describing unexplained variations (Fig. 6A). Subsequent analyses (Bayesian R-squared) [38] highlighted that approximately 56% of variation in the weekly number of clinical malaria cases in Ethiopia is explained by the model with all components. Within the explained component, 55% is due to fixed effects.

**Fig. 5:**
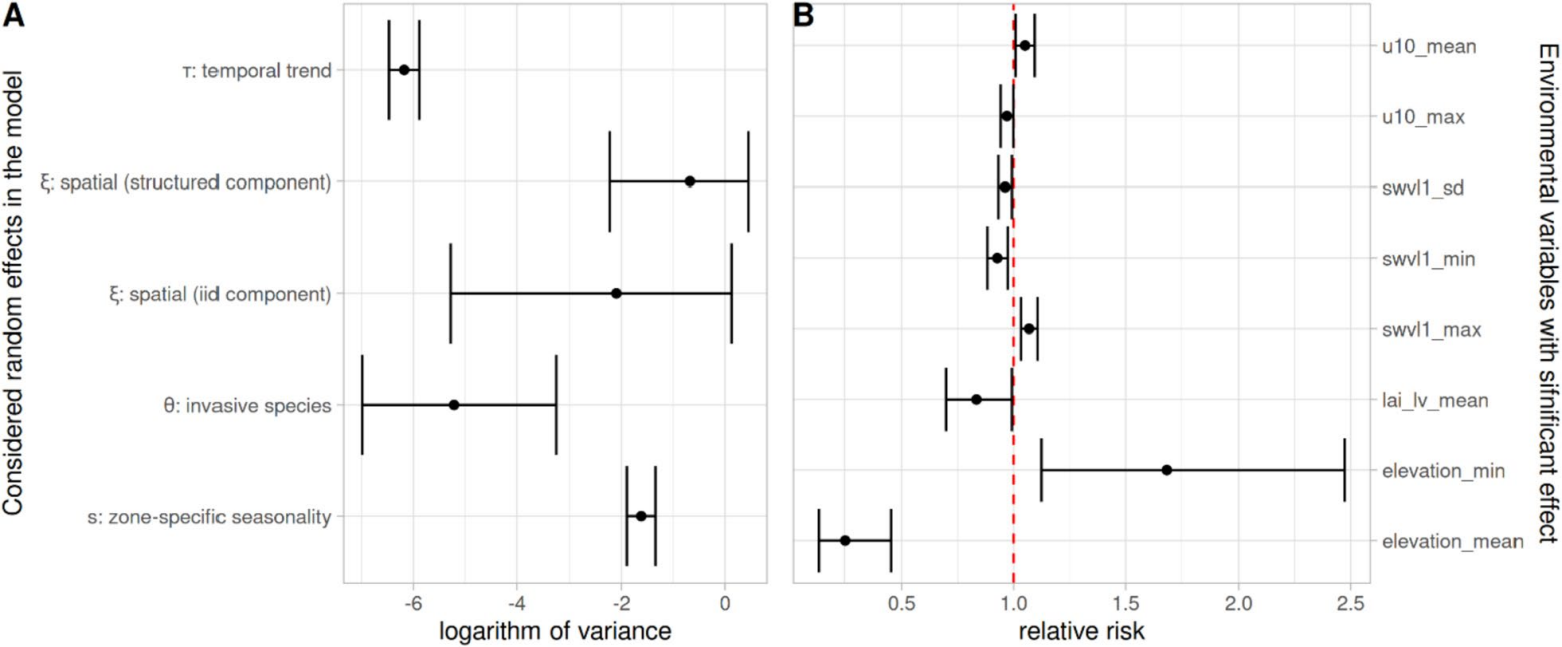
Estimated means and their corresponding 95% credible intervals of logarithms of variance for all random effect terms within the considered model: spatial random effect ξ_i, overall temporal random effect τ_t, zone-specific seasonal random effect s_(i,t) and the presence or absence of invasive species ν_(i,t) (**A**). Forest plot of the estimated means and their corresponding 95% credible intervals of statistically significant coefficients for environmental factors in the considered model for clinical malaria cases in Ethiopia (**B**)

**Fig. 6:**
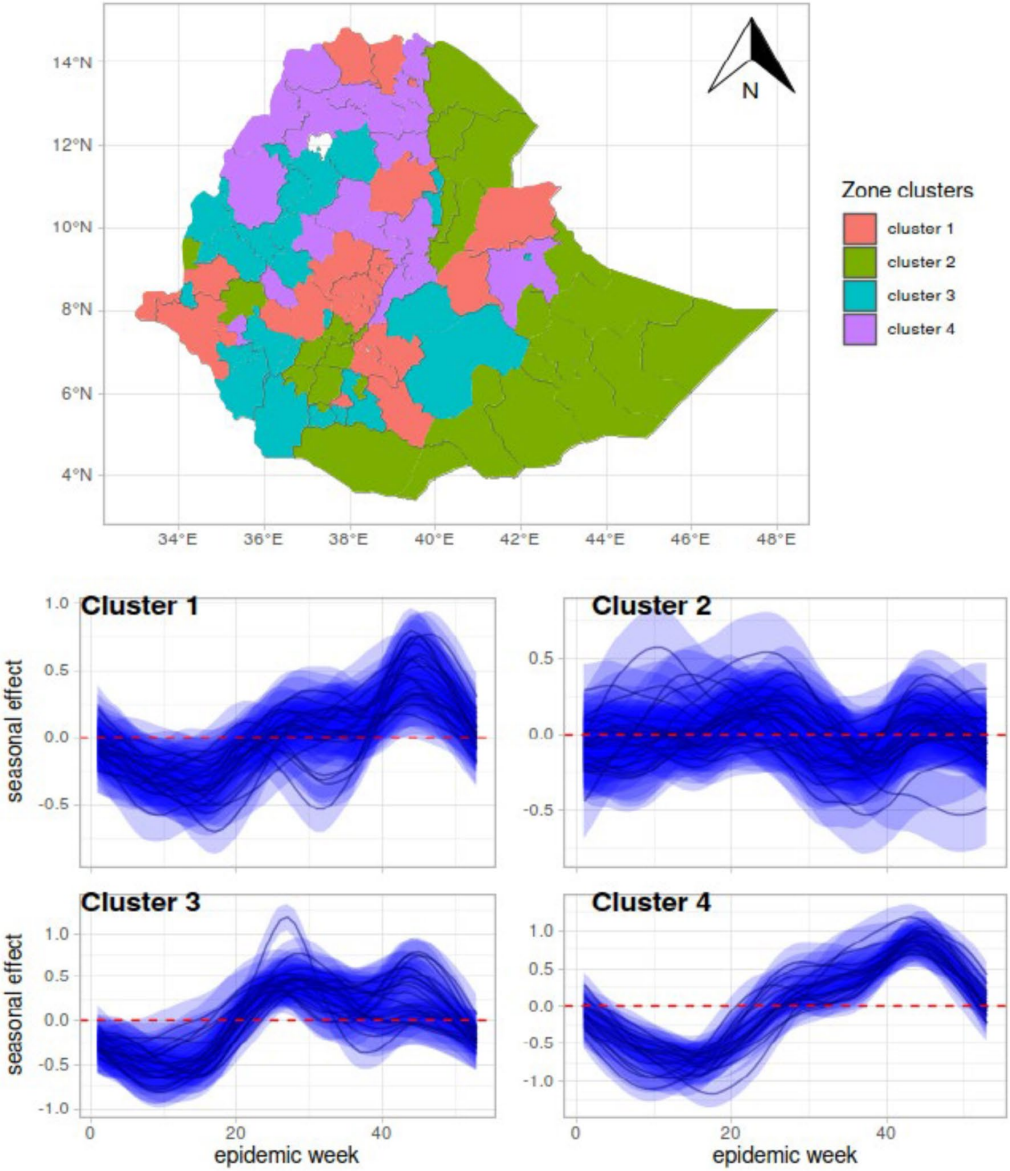
Hierarchical clustering of Ethiopia’s zones based on the similarity of their estimated zone-specific seasonal random effects in 4 clusters (top panel) with the posterior mean and 95% confidence intervals of seasonal effects displayed for each cluster (bottom panels)

From the fixed effects variables described in Table 1, seven were found significantly associated with weekly malaria variation over the zones (Fig. 6B). In particular the (1) horizontal eastly wind speed at a height of up to 10 m (u10): Higher average wind speed within a zone is associated with a slight (5%) increase in malaria risk. Surface soil moisture (swvl1), (2) the maximum amount of water held in the top soil layer (0–7cm) across each zone significantly increases the risk of malaria by 7%. Conversely, both the standard deviation and minimum of swvl1 within each zone showed a decrease of around 4% and 7% in malaria risk respectively, with each unit increase, (3) Leaf area index for low vegetation: The mean leaf area index for low vegetation decreases malaria risk by 17% and (4) Elevation: higher minimum elevation is associated with 69% increase in malaria risk while mean elevation is associated with 75% decrease (Fig. 5).

### Observed clustering and seasonality is unexplained by environmental factors

The estimated zone-specific seasonal random effects indicated that there is residual seasonal variation (the part of seasonality not explained by environmental factors) across both space and time (Supplementary Fig. 1). Some zones, like Kilbati, do not show a clear seasonal pattern beyond what environmental factors can explain, while others exhibit distinct cycles, with winter declines and summer increases. A hierarchical cluster analysis using a complete linkage method [36] identified four geographical clusters (Fig. 6, top panel) with local seasonality not explained by environmental variables (Supplementary Fig. 2). Zones in Cluster 4 demonstrate consistent seasonal patterns, with significant declines in March and April and significant peaks in October and November. In contrast, zones in Cluster 2 and, to some extent, those in Cluster 1 showed no consistent seasonality. Zones in Cluster 3, however, consistently peak in June. In terms of malaria risk representation, zones in Cluster 3 have a significantly higher malaria risk [95% Confidence interval (CI): 10.8–15.9] than Cluster 2 (95% *CI:* 4.96–7.37). Zones in clusters 1 and 4 tend to have an overall medium risk (Supplementary Table 3). The “[(95% Confidence interval (CI): 10.8–15.9]" should change to: “95% confidence interval (*CI*): 10.8–15.9]”.

## Discussion

### Progress in malaria control and elimination efforts in Ethiopia has stalled since 2021

This study revealed that malaria transmission in Ethiopia exhibited a cyclical seasonal pattern and a declining long-term temporal trend between 2013 and 2018, followed by increases in transmission up to 2021 similar to those observed in the WHO African Region [7]. The models identified a higher concentration of clinical malaria cases in the western and northwestern zones bordering countries.

### Approximately 50% of variability in malaria trends over the last decade in Ethiopia could be attributed to environmental factors

The observed lower variance in overall temporal effects implies that large-scale temporal variations play a lesser role. Fixed effects (represented by the selected seven environmental variables), account for approximately 55% of explained variance, and the larger variances in zone-specific seasonal and spatially structured random effects highlight the importance of local variations in understanding the remaining unexplained variations in malaria risk (approximately 45%). While environmental variables like easterly horizontal wind speed at a height of 10 m, surface soil moisture, and higher minimum elevation were associated with increased malaria risk, lower mean leaf area index of low vegetation and mean elevation were associated with decreased risk. However, a substantial amount of unexplained seasonal variation across space and time remained, as captured by zone-specific seasonal random effects, and tended to cluster in regions that are not always geographically contiguous. The significantly higher risk observed in the northwest and lower risk in the southwestern part of Ethiopia (Figs. 3 and 4) is potentially due to a complex interplay of factors. The model outputs are consistent with Ethiopian malaria transmission patterns, which are seasonal, inter-annual and spatially heterogeneous, except in the low-lying southwestern border areas experiencing perennial transmission [13, 14]. Factors influencing mosquito breeding, parasite survival, and consequently malaria transmission, including environmental factors such as temperature and rainfall [39], might play important roles in the observed variation. Variation in malaria seasonality across zones, as well as clustering following similar patterns (Figs. 4 and 6), is consistent with previous studies that documented significant geographical heterogeneity in malaria transmission. Regional and subregional spatial clustering and the presence of hotspots were attributed to environmental and socioeconomic factors. In the northwest, from 2009 to 2010 focal upsurges in malaria cases with spatiotemporal patterns were reported [40]. Low malaria risk in eastern zones compared to the northwestern zones might be due to variations in rainfall patterns and seasonality as observed elsewhere [39]. The overall trend in our risk map (Fig. 4) aligns with the Ethiopian national malaria risk stratification map [14], showing a relatively higher burden in the western and northwestern parts of the country which is also consistent with non-epidemic years [13]. Factors that could have a bearing on the nationwide upsurge in addition to the environmental and socioeconomic variation might be the consequences of long and widespread internal conflict and population displacement [12] as well as the COVID-19 pandemic [10] which disrupted the health system. Therefore, the adverse effects of the interplay between climatic anomalies and conflict-associated displacement in populations at high risk of neglected tropical diseases is highlighted as a strategic research focus [12]. The other potential risk factor which could have contributed to the changes in clinical malaria trends is *An. stephensi*, reported mainly in east and southeastern Ethiopia [8, 41] but not in others [42]. We need to be cautious but our model identified low malaria in eastern Ethiopia where *An. stephensi* is well-established [11, 19].

In our model, a horizontal easterly wind speed at a height of ten meters above the Earth's surface was associated with a 5% increase in malaria risk. This might be explained by the fact that wind can assist mosquitoes in host-seeking [43] and wind-borne migration of mosquitoes [44]. Importantly, the direction of the wind from villages to the breeding habitats has been linked with an increased population size of mosquitoes compared to the opposite wind direction [45]. However, wind can also generate waves on the water surface, which can be fatal to the aquatic life stage of mosquitoes in large reservoirs compared to small rain-made breeding habitats [46].

Surface soil moisture (the volume of water in the first layer (0–7 cm) of surface soil) is a good predictor of malaria risk in this study, as indicated elsewhere [47]. Previous studies from Nigeria [48] and Uganda [49] have shown that the vegetation index was an important contributor to malaria risk. Conversely, the mean leaf area index for low vegetation decreased malaria risk by 17% in our study. Importantly, a decrease in elevation (altitude) was significantly associated with an increased relative risk of malaria in our study, while the average higher elevation was not. However, warmer years could promote malaria transmission at higher altitudes in Ethiopia and Colombia [50].

### Observed local clustering and seasonality is unexplained by environmental factors

Our study also suggests the existence of local (small-scale) spatial and temporal variations more important than large-scale variations that could shape Ethiopia's malaria risk distribution (Fig. 6A and B). This is evident from the substantial variance in zone-specific seasonal effects, highlighting how seasonal fluctuations within zones influence overall risk. Potentially local human behaviours and mosquito ecology [51] as well as socioeconomic conditions [52] might vary across zones and seasons, impacting transmission dynamics. Importantly, significant variation in the spatially structured effect suggests that local spatial patterns matter. Shared patterns between neighbouring zones could contribute to this spatial clustering [53]. Importantly, within each cluster, it is likely that zones share common factors that were not considered in the model such as mosquito vector population, human behavioral factors, differences in healthcare structure, and malaria interventions. biological threats such as drug and diagnostic-resistant *P. falciparum* reported in different parts of Ethiopia [8, 9].

Our study revealed the spatial and temporal trends of malaria across Ethiopian zones. The local variation in transmission emphasizes the need for tailored interventions across zones but also their potential as sources of epidemics [54]. We acknowledge that the findings should be cautiously interpreted. The inherent incompleteness of the PHEM data and the use of different time scales with environmental data might have resulted in an underestimation of risks. Considering more risk factors, socioeconomic and near-surface temperature, and considering a time lag with nonlinear modelling might have improved the outcome.

## Conclusions

Our findings underline the importance of environmental factors, accounting for 55% of the variability explained by our model, as drivers of the spatiotemporal distribution of malaria risk in Ethiopia. The local clustering and seasonality unexplained by environmental factors call for further exploration into tailored focal responses. The presence of a sizable proportion (45%) of risk factors accounting for changes in the trends of clinical malaria remain unexplained by our model; information about biological threats (vector dynamics; change in insecticide resistance and/or biting and resting behavior, occurrence of drug and diagnostic resistance in *Plasmodium* populations) and years in the grip of internal conflict could have played important roles. This study showed that reduced effectiveness of malaria control efforts in a country after years of progress towards elimination highlights the need for multi-sectoral coordination, and a localised and coordinated response beyond the health system.

## Supplementary Information

Additional file 1

## Data Availability

The data underlying this article will be shared upon reasonable request to the corresponding author.
