# Supplementary material for: Waning success: a 2013–2022 spatial and temporal trend analysis of malaria in Ethiopia
Source: Infect Dis Poverty. 2024 Dec 9;13:93. doi: 10.1186/s40249-024-01259-4 (PMC11626767; doi:10.1186/s40249-024-01259-4)
Supplement: Supplementary file 1 — Additional file 1 [file 40249_2024_1259_MOESM1_ESM.docx]

**Supplementary files**

**Supplementary Table 1.** Summary statistics of the weekly number of clinical malaria cases in zones of Ethiopia aggregated annually and across the whole study period. The exposed population for each year is calculated as the cumulative population size of zones with non-missing data on clinical malaria cases.

| **Year** | **Mean** | **Standard deviation** | **Min** | **1^st^ quartile** | **Median** | **3^rd^ quartile** | **Max** | **Exposed Population** |
| --- | --- | --- | --- | --- | --- | --- | --- | --- |
| 2013 | 823.7 | 1090.9 | 0 | 127 | 366 | 1167.75 | 12622 | 80,940,353 |
| 2014 | 537.0 | 807.3 | 0 | 110 | 257 | 694 | 11505 | 83,125,742 |
| 2015 | 428.5 | 753.4 | 0 | 83 | 185 | 409 | 10815 | 85,370,137 |
| 2016 | 430.1 | 693.9 | 0 | 83 | 190 | 439.75 | 8206 | 89,556,108 |
| 2017 | 308.7 | 529.3 | 0 | 60 | 129 | 309 | 6841 | 92,648,115 |
| 2018 | 238.5 | 404.4 | 0 | 42 | 92 | 263 | 5571 | 95,503,848 |
| 2019 | 342.3 | 539.4 | 0 | 50.5 | 138 | 373.5 | 4974 | 98,082,452 |
| 2020 | 333.9 | 561.9 | 0 | 52 | 155 | 356.75 | 6607 | 101,845,634 |
| 2021 | 300.3 | 548.2 | 0 | 42 | 128 | 311 | 8122 | 105,068,112 |
| 2022 | 613.6 | 1127.4 | 0 | 71 | 215 | 683.75 | 15913 | 102,011,002 |
| 2023 | 656.1 | 891.2 | 0 | 85 | 317 | 765 | 5212 | 104,765,299 |
| Overall | 432.8 | 758.1 | 0 | 65 | 170 | 456 | 15913 | 105,068,112 |

**Supplementary Table 2**. Population in 2022, the number of zones, and summary statistics of the weekly counts of clinical malaria cases for each region in Ethiopia.

| **Region** | **Population in 2022** | **Number of zones** | **Mean** | **Standard deviation** | **Min** | **1^st^ quartile** | **Median** | **3^rd^ quartile** | **Max** |
| --- | --- | --- | --- | --- | --- | --- | --- | --- | --- |
| Addis Ababa | 3,859,999 | 1 | 71.8 | 34.7 | 0 | 47 | 67 | 91 | 210 |
| Afar | 2,033,002 | 5 | 278.2 | 192.2 | 0 | 149 | 251 | 367 | 1594 |
| Amhara | 22,876,991 | 12 | 936.9 | 1415.7 | 0 | 149 | 362 | 1155 | 15913 |
| Benishangul-Gumuz | 1,218,000 | 4 | 961.6 | 920.9 | 0 | 170 | 707.75 | 1490.25 | 5571 |
| Dire Dawa | 535,000 | 1 | 42.3 | 95.3 | 1 | 11 | 19 | 37 | 1128 |
| Gambela | 508,004 | 4 | 440.1 | 492.7 | 0 | 136 | 243 | 581 | 4333 |
| Harari | 276,000 | 1 | 91.7 | 107.1 | 1 | 17.75 | 48 | 117.25 | 579 |
| Oromia | 39,926,505 | 20 | 249.9 | 387.7 | 0 | 48 | 121 | 278 | 5431 |
| SNNP | 13,763,888 | 17 | 437.8 | 549.5 | 0 | 65 | 207 | 634 | 4645 |
| Sidama | 4,141,905 | 1 | 929.7 | 1113.1 | 0 | 283.75 | 469 | 981 | 7396 |
| Somali | 6,602,612 | 11 | 102.0 | 76.7 | 0 | 41 | 82 | 151 | 719 |
| South West | 3,587,210 | 6 | 303.9 | 429.9 | 0 | 67 | 172 | 350.5 | 4219 |
| Tigray | 5,738,996 | 7 | 573.8 | 749.0 | 0 | 52 | 215 | 879 | 5829 |


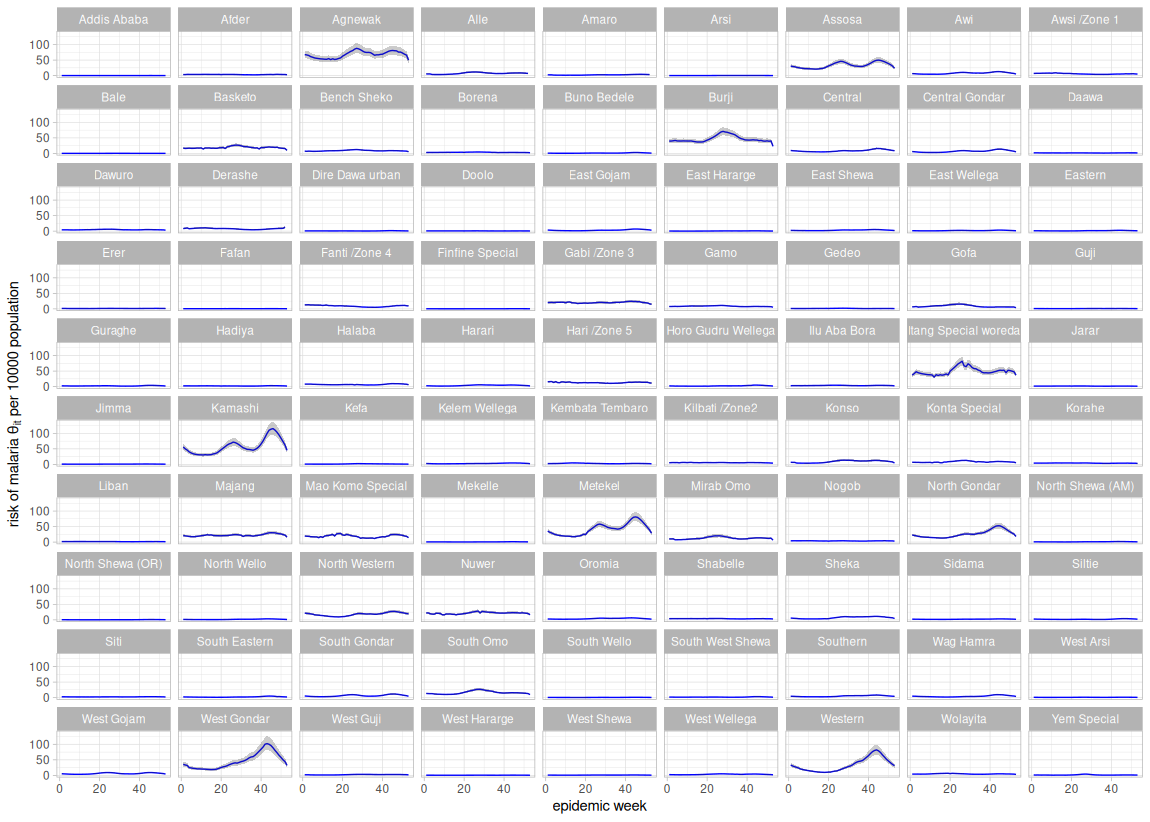


**Supplementary figure 1.** Mean (solid line) and 95% credible interval (grey shading) of the estimated risk of malarial per 10000 population for each week in different zones of Ethiopia.


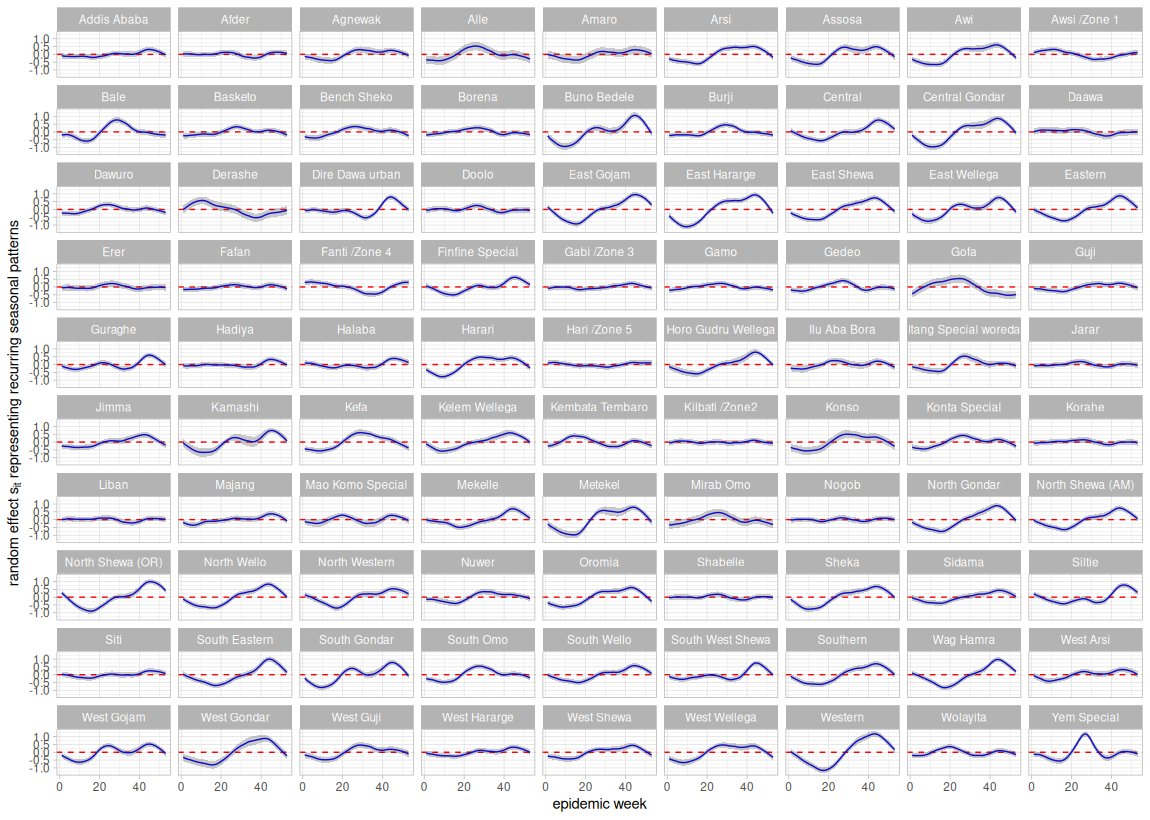


**Supplementary figure 2.** Mean (solid line) and 95% credible interval (grey shading) of the temporal random effect representing weekly recurring seasonal patterns in different zones of Ethiopia.

**Supplementary Table 3.** The posterior mean of malaria risk for each cluster with a list of zones in Ethiopia

| Zone clusters | Member zones (region) | Posterior mean of risk | 95% Credible Interval of risk |
| --- | --- | --- | --- |
| Cluster 1 | 1 Addis Ababa (Addis Ababa)  2 South Wello (Amhara)  3 Dire Dawa urban (Dire Dawa)  4 Agnewak (Gambela)  5 Majang (Gambela)  6 Nuwer (Gambela)  7 Finfine Special (Oromia)  8 Guji (Oromia)  9 Jimma (Oromia)  10 Kelem Wellega (Oromia)  11 South West Shewa (Oromia)  12 West Arsi (Oromia)  13 West Hararge (Oromia)  14 West Shewa (Oromia)  15 Amaro (SNNP)  16 Guraghe (SNNP)  17 Siltie (SNNP)  18 Sidama (Sidama)  19 Siti (Somali)  20 Central (Tigray)  21 Mekelle (Tigray)  22 North Western (Tigray) | 7.34 | (6.05 , 8.81) |
| Cluster 2 | 1 Awsi /Zone 1 (Afar)  2 Fanti /Zone 4 (Afar)  3 Gabi /Zone 3 (Afar)  4 Hari /Zone 5 (Afar)  5 Kilbati /Zone2 (Afar)  6 Mao Komo Special (Benishangul-Gumuz)  7 Borena (Oromia)  8 Ilu Aba Bora (Oromia)  9 Basketo (SNNP)  10 Derashe (SNNP)  11 Gamo (SNNP)  12 Gedeo (SNNP)  13 Gofa (SNNP)  14 Hadiya (SNNP)  15 Halaba (SNNP)  16 Kembata Tembaro (SNNP)  17 Wolayita (SNNP)  18 Afder (Somali)  19 Daawa (Somali)  20 Doolo (Somali)  21 Erer (Somali)  22 Fafan (Somali)  23 Jarar (Somali)  24 Korahe (Somali)  25 Liban (Somali)  26 Nogob (Somali)  27 Shabelle (Somali)  28 Dawuro (South West) | 6.07 | (4.96, 7.37) |
| Cluster 3 | 1 Awi (Amhara)  2 Oromia (Amhara)  3 South Gondar (Amhara)  4 West Gojam (Amhara)  5 Assosa (Benishangul-Gumuz)  6 Kamashi (Benishangul-Gumuz)  7 Itang Special woreda (Gambela)  8 Harari (Harari)  9 Arsi (Oromia)  10 Bale (Oromia)  11 East Wellega (Oromia)  12 West Guji (Oromia)  13 West Wellega (Oromia)  14 Alle (SNNP)  15 Burji (SNNP)  16 Konso (SNNP)  17 South Omo (SNNP)  18 Yem Special (SNNP)  19 Bench Sheko (South West)  20 Kefa (South West)  21 Konta Special (South West)  22 Mirab Omo (South West) | 13.1 | (10.8, 15.9) |
| Cluster 4 | 1 Central Gondar (Amhara)  2 East Gojam (Amhara)  3 North Gondar (Amhara)  4 North Shewa (AM) (Amhara)  5 North Wello (Amhara)  6 Wag Hamra (Amhara)  7 West Gondar (Amhara)  8 Metekel (Benishangul-Gumuz)  9 Buno Bedele (Oromia)  10 East Hararge (Oromia)  11 East Shewa (Oromia)  12 Horo Gudru Wellega (Oromia)  13 North Shewa (OR) (Oromia)  14 Sheka (South West)  15 Eastern (Tigray)  16 South Eastern (Tigray)  17 Southern (Tigray)  18 Western (Tigray) | 10.5 | (8.56, 12.7) |
